# Effects of bone marrow cell transplant on thyroid function in an I^131^-induced low T4 and elevated TSH rat model

**DOI:** 10.1186/1477-5751-6-1

**Published:** 2007-01-18

**Authors:** Gustavo E Guajardo-Salinas, Juan A Carvajal, Ángel A Gaytan-Ramos, Luis Arroyo, Alberto G López-Reyes, José F Islas, Beiman G Cano, Netzahualcoyótl Arroyo-Currás, Alfredo Dávalos, Gloria Madrid, Jorge E Moreno-Cuevas

**Affiliations:** 1 Cell Therapy Laboratory, ITESM School of Medicine, 3000 Ave. Morones Prieto, Monterrey, NL 64710, México

## Abstract

**Background:**

We developed a study using low dose radioactive iodine creating an animal model of transient elevation of thyroid stimulating hormone (TSH). Male derived bone marrow cells were transplanted to asses their effect on thyroid function and their capability to repair the thyroid parenchyma.

**Results:**

At 40 an 80 days after I^131 ^treatment, the study groups TSH and T4 serum values both increased and decreased significantly respectively compared to the negative control group. Eight weeks after cell transplantation, neither TSH nor T4 showed a significant difference in any group. The mean number of SRY gene copies found in group I (Left Intracardiac Transplant) was 523.3 and those in group II (Intrathyroid Transplant) were only 73. Group III (No Transplant) and IV had no copies. Group I presented a partial restore of the histological pattern of rat thyroid with approximately 20% – 30% of normal-sized follicles. Group II did not show any histological differences compared to group III (Positive control).

**Conclusion:**

Both a significant increase of TSH and decrease of T4 can be induced as early as day 40 after a low dose of I^131 ^in rats. Restore of normal thyroid function can be spontaneously achieved after using a low dose RAI in a rat model. The use of BM derived cells did not affect the re-establishment of thyroid function and might help restore the normal architecture after treatment with RAI.

## Background

In the past decade, there have been many reports that provide evidence about the multi-lineage potential of stem cells, [1-3]. Adult stem cells have shown board plasticity that has allowed treatment of heart, liver and principally, blood related disorders, [1,4]. This plasticity and the availability of adult bone marrow stem cells have made them a very promising source for research and clinical treatments.

The use of Iodine-131 (I^131^) to treat patients with hyperthyroidism, Grave's disease, has common side effects such as hypothyroidism that can be permanent [5-12]. In children and adolescent cases, hypothyroidism have been reported in 50 to 95% of patients at 1 year after treatment with Iodine-131 [13-15]. Most studies on the effects of Radioactive Iodine (RAI) on thyroid function conclude that this change is permanent, which is why many patients remain hypothyroid and have the physiological need to use supplemental thyroid hormones for the rest of their lives. Previous attempts to restore the thyroid function have been made by the removal and cryopreservation of thyroid tissue before treatment with I^131^, [16]. Stem cells might offer a new therapeutic approach, instead of using cryopreserved thyroid tissue, but it has not been studied yet.

It is a well-known fact that bone marrow cells (BM) can give rise to hepatic oval cells, hepatocytes, cholangiocytes, skeletal-muscle cells, astrocytes, and neurons. Nonetheless, no studies report the use of BM cells in endocrine disorders like hypothyroidism [2,3]. To investigate whether adult bone marrow cells are able to aid in the reconstitution of thyroid parenchyma, we conducted a study based on the implantation of male derived BM cells into thyroid gland of female rats previously treated with RAI to induce an iatrogenic state of low T4 and elevated TSH, [17-20]. It has been shown that the embryonic stem cells can differentiate in vitro like cells under the stimuli of TSH, [21]. Our study, using low dose RAI, will create an animal model of transient elevation of TSH and with transplantation of male derived BM cells, it is possible to asses their effect on thyroid function, and if they are able, to participate in the repair of thyroid parenchyma.

## Results

### Serum total T4

**Figure 1:**
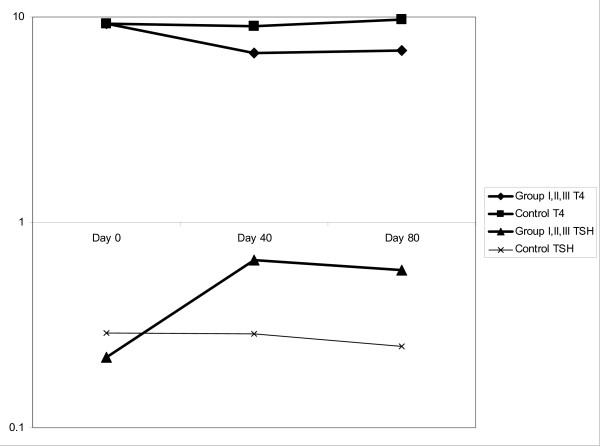
**TSH and T4 concentration on Day 0, 40 and 80 after RAI**. TSH and T4 serum levels of study group (n = 15) compared to control group (n = 5). There was statistical difference since day 40 remaining at day 80 when all transplants where done.

Serum levels of Total T4 decreased significantly by day 40 and remained as such, until day 80, in the study group. The study group mean was 6.67 ug/dL(SD 1.49) and 6.84 ug/dL(SD 2.31) on days 40 and 80 respectively versus 9.02 ug/dL(SD 1.66) and 9.68 ug/dL(SD 1.59) of control group (p < .01). See Fig. (1).

**Figure 2:**
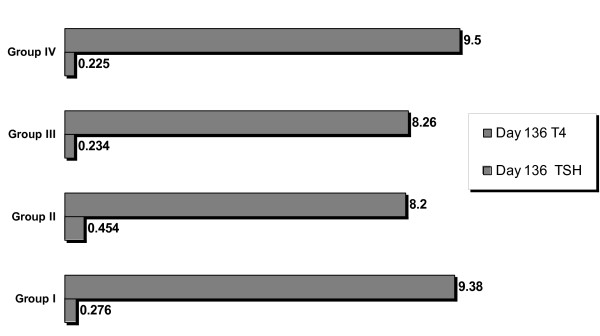
**TSH and T4 concentrations 8 weeks after transplantation of stem cells**. TSH and T4 serum levels of all groups 8 weeks after BM derived cells transplantation. All concentrations of T4 are very similar in all groups with no statistical difference between them. TSH concentration of group II although high compared to other groups had no significant difference. TSH was repeated for group II at 10 weeks after transplantation and the mean was 0.140 μIU/mL.

On day 80, the study group was divided into Group I: Intraventicular Transplant, Group II: Local Thyroid Transplant and Group III: No Transplant. By day 136 (8 weeks after the transplant) the serum levels of T4 were 9.38 ug/dL (SD 2.63) in group I, 8.2 ug/dL (SD 0.92) in group II, 8.26 ug/dL (SD 0.92) in group III and 9.5 ug/dL(SD 1.41) in the negative control group, with no significant difference between them. See Fig. (2).

### Serum TSH

TSH serum levels were elevated significantly by day 40 and remained in the study group until day 80. The study group mean was 0.650 μIU/mL (SD 0.39) and 0.588 μIU/mL (SD 0.40) on day 40 and 80 respectively versus 0.287 μIU/mL (SD 0.12) and 0.250 μIU/mL (SD 0.12) in the control group (p < .05). See Fig. (1). On day 136, 8 weeks after the transplant, the serum levels of TSH were 0.276 μIU/mL (SD 0.12) in group I, 0.454 μIU/mL (SD 0.17) in group II, 0.234 μIU/mL (SD 0.04) in group III and 0.226 μIU/mL (SD 0.10) in group IV, with no significant difference between them.

Although no significant difference was found among all studied groups, Group II TSH was measured again on week 10 and the results established that it had reached basal levels of 0.140 μIU/mL. See Fig. (2).

When the basal serum values of T4 and TSH on day 0 were compared to those of both day 40 and day 80, a significant elevation of TSH (p < .05) and a significant decrease of T4 (p < .05) were found.

### Real time PCR for SRY segment after reconstitution of thyroid function

**Figure 3:**
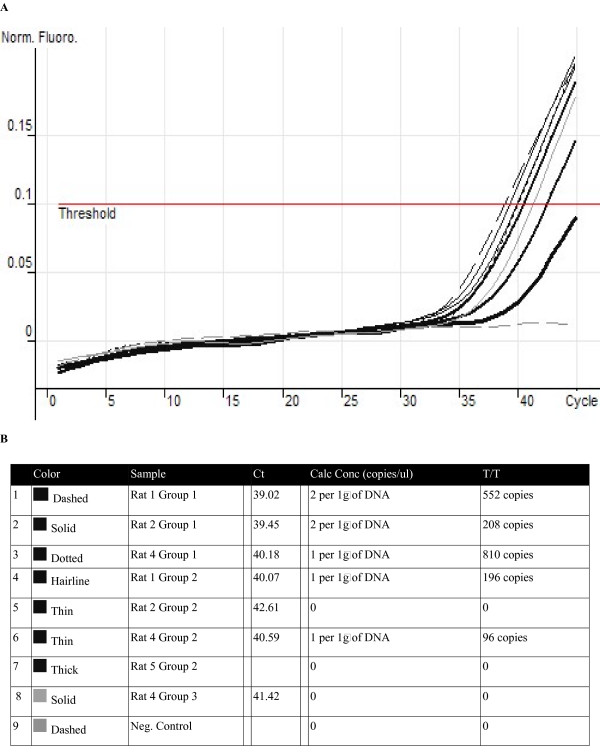
**SRY Amplification using Real Time PCR**. **A**. Amplification curves of all samples. The negative control did not have any amplification. Some DNA samples from group 1 and group 2 were not included in the analysis due to low quality of the DNA extracted. **B**. Quantitative Analysis Report. Amplification after cycle 41 was taken as background DNA and no copies where reported in the Rotor-Gene 6 software. Ct = Cycle Threshold, Calc Conc = Calculated Concentration, T/T = Total Copies in the Thyroid Gland (Assuming both thyroid lobes were the same size).

The mean number of copies of the SRY gene found in the thyroid for Group I was 523.3, and Group II had a mean of 73 copies. Group III and IV had an amplified DNA background but no reported copies of the SRY gene when quantification analysis was completed. See Fig. (3).

### Histological morphology

**Figure 4:**
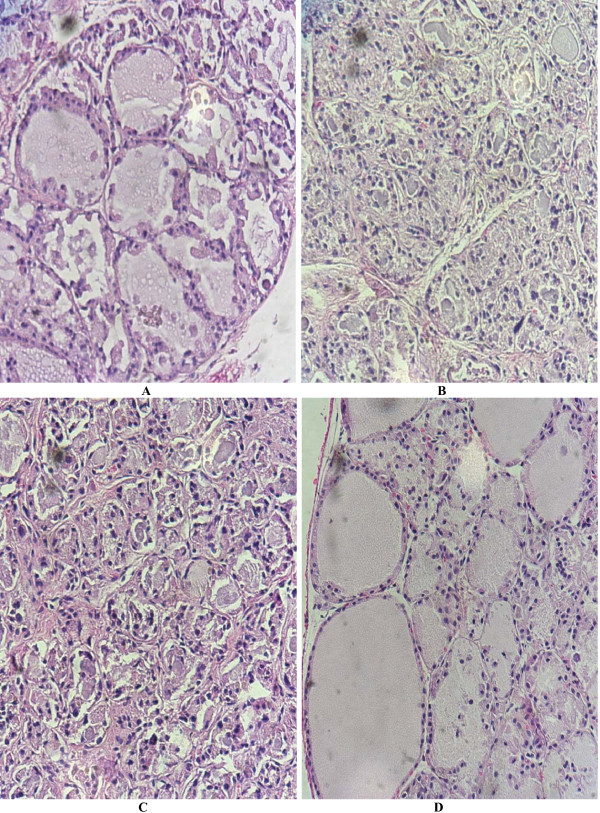
**Microscopic appearance of rat thyroid after bone marrow cell transplant**. **A**. Group I H + E. 10 weeks after left intra ventricular transplantation. **B**. Group II H + E. 10 weeks after direct intra thyroid transplantation. **C**. Group III H+E. (Positive Controls). **D**. Group IV H + E. (Negative Controls).

Group I showed a partial restore of the normal histological pattern of rat thyroid with approximately 20%–30% of normal-sized follicles. Group II did not show any histological difference compared to Group III (Positive Control); both had small follicles with almost no colloid inside. Group IV (Negative Control) showed normal rat thyroid histology. See Fig. (4).

## Discussion

We induced a transient elevation of TSH and a transient decrease in serum levels of Total T4 using a low dose Iodine-131 IP. During the transient elevation of TSH we transplanted BM cells from a male donor using two different approaches (left ventricle puncture and Intrathyroid infusion). We found that after low dose I-^131 ^injection, significant elevation of TSH and decrease of T4 were obvious since day 40 and remained at least until day 80 when transplantation procedures were done. After 8 weeks we measured TSH and T4 finding no significant changes when compared to the negative control group; this procedure proved that the thyroid function was restored to normal. In contrast to previous studies where all study rats were killed after they significantly reduced thyroid function, both TSH and T4 can be restored to normal levels in a rat model on day 136.

Although evidence that BM derived cell can be implanted in the thyroid gland and remain at least 10 weeks later was found, the results shown by the Real Time PCR analysis confirmed the presence of only a small amount of male derived cells. Therefore, with this evidence it is possible to elucidate that bone marrow stem cells got implanted into the thyroid parenchyma but did not significantly help to reconstitute the thyroid after a partial damage with Iodine-131. Furthermore the recovery time of normal TSH levels was no different than that of group I and group III, and there was a delay in the recovery of normal thyroid function in group II in comparison to both groups I and III that may be caused by the inflammation induced by the direct infusion of the cells into the thyroid parenchyma. The first measurement of TSH and T4 after the transplant was done 8 weeks later and at this time both group I and III had normal levels of both hormones, but we cannot determine accurately which group first regained thyroid function. Thusly, further studies are needed to resolve this matter, and even though the presence of Y chromosome on the thyroid gland of female rats was found, it is also not possible to know which of these cells differentiated into follicular cells. An interesting fact worth mentioning is that the rats, group I, that showed a higher number of copies of the Y chromosome, also presented a partial restore of the normal histological architecture of the thyroid.

## Conclusion

Both a significant increase of TSH and decrease of T4 can be induced as early as day 40 after a low dose of I^131 ^in rats. Restore of normal thyroid function can be spontaneously achieved after using a low dose RAI in a rat model. The use of BM derived cells did not affect the re-establishment of thyroid function and might help restore the normal architecture after treatment with RAI.

## Methods

### Animals

Twenty female Wistar rats of 200–210 g were maintained on food and water with no restrictions. No attempt was made to restrict iodine intake. Fifteen rats were injected IP with 150 uCi diluted to 0.5 ml of saline and five rats were injected IP with only 0.5 ml of saline. Diagnosis of what we called "transient hypothyroidism" (TH) was based on significant elevation of serum TSH and serum Total T4 decrease after I^131 ^followed by recovery of normal TSH and T4. Blood samples were taken by orbital puncture under diethyl-ether anesthesia. All samples were collected between 14:00–18:00 hrs on day 0, 40, 80, and 136. All rats were sacrificed after 10 weeks of transplantation using a lethal dose (100 mg /100 gr) of IP pentobarbital (Anestesal^® ^63 mg/1 ml).

### Serum hormone levels

We measured serum Total T4 using a human Enzyme Immunoassay (EIA) kit (DSL^®^) and serum TSH using a human Enzyme-linked Immunoassay (ELISA) kit (DSL^®^); all determinations were done at least twice.

### Bone marrow cell extraction and culture

Under a lethal dose of anesthesia (Pentobarbital 100 mg/100 gr) both femurs and tibia were dissected from a 10 week old male Wistar rat and were washed thoroughly to avoid possible contamination with cells outside the bone marrow. Then both ends of each femur were cut and the bone marrow was washed out with phosphate-buffered saline (PBS). Cells were then cultured using DMEM F12 supplemented with 10% KO (Knock out Serum) and 1% antibiotic solution (5,000 U.I/ml Penicillin-5,000 ug/ml Streptomycin) at 37 C with CO2 5% and 95% humidity.

### Transplants

All transplants were done on day 80 after I^131 ^when the study animals reached a significant elevation of TSH and a significant decrease of T4 compared to the basal levels and with the control group levels, then they were divided into three groups. Group I rats (n = 5) were transplanted with 1 × 10^6 ^BM cells suspended in 1 ml of normal saline via Intracardiac left ventricle puncture using profound anesthesia with IP pentobarbital (40 mg/100 gr). Group II rats (n = 5) were transplanted with 1 × 10^6 ^BM cells via intra thyroid puncture under direct vision after neck dissection using profound anesthesia with IP pentobarbital (40 mg/100 gr). Group III rats (n = 5) were used as "TH" positive controls and received no treatment. Group IV rats (n = 5) were used as "euthyroid" negative controls and did not received I-^131 ^or BM derived cells.

### Real Time Polymerase Chain Reaction (PCR) for the sex determining region gene (SRY)

Rats were sacrificed under lethal dose of anesthesia (100 mg/100 gr pentobarbital) 10 weeks after transplantation of SRY positive male BM cells and the right lobe of the thyroid gland was surgically removed.

We used a commercial DNA extraction kit (Wizard^®^) to extract thyroid DNA and then, Real Time PCR was done using Platinum^® ^qPCR SuperMix UDG with SRY gene specific lux primer (Invitrogen^® ^Mexico) to asses the presence of male DNA (SRY gene). PCR reaction solution: 10 ul qPCR SuperMix UDG, 8 ul distilled water DNAse RNAse free, 1 μg of DNA for each sample.

Real Time-PCR program (Rotor Gene 3000) was run as: 50 C for 2 min: 1 cycle; 95 C for 2 min, 1 cycle; 60 C for 30 sec, 95 C for 15 sec; 40 cycles in all. We used a standard curve for quantitative analysis. Standard DNA was provided by Invitrogen^®^, at a concentration of 10^9 ^copies per ul.

Primer Forward Sequence: 5'-caccttcCCTTTCCCACAGATAAGAAGG5G'-3'

Reverse Sequence: 5'-CAATGGGCTGGGAAGAATTAACA-3'. The PCR product size was 66 bp.

### Histological cuts

One lobe of the thyroid gland of each rat was obtained and fixed in 4% paraformaldehyde overnight and stained using hematoxylin-eosin (H&E). Histological cuts were examined under standard light microscopy (Olympus^®^) at 10×, 20× and 40×.

### Statistical analysis

Statistical analysis between groups was done with SPSS^® ^for Windows^® ^Version 12.0 using t-student test. Microsoft Excel^® ^2003 was used to create figures.

## Competing interests

The author(s) declare that they have no competing interests.

## Authors' contributions

GEGS: Conceived and participated in the design of the study, helped and supervised all tests and analysis during the study. Also performed the BM derived cells transplants. JAC: carried out the ELISA and EIA tests AAGR: carried out the blood extraction and cell cultures LA: participated in the design and helped with serum extraction AGLR: carried out the real time PCR and performed the BM cell extraction. JFI: carried out the I^131 ^administration to the study rats. BGC: carried out the DNA extraction. NAC: carried out organ extraction and participated in the DNA extraction and in the real time PCR. AD: performed the statistical analysis and participated in the writing of this paper. GM: participated in the organ extraction and performed histological cuts and histological analysis. JEMC: Supervised and participated in the design of the study and the writing of this paper.

## References

[B1] Körbling M, Anderlini P Peripheral blood stem cell versus bone marrow allotransplantation: does the source of hematopoietic stem cells matter?. Blood.

[B2] Kôrbling Martin, Estrov Zeev (2003). Adult Stem Cells for Tissue Repair – A new Therapeutic Concept?. N Engl J Med.

[B3] Odorico Jon S, Kaufman Dan S, Thomson James A (2001). Multilineage Differentiation from Human Embryonic Stem Cell Lines. Stem Cells.

[B4] Piano MR, Carrigan TM (2003). Cellular cardiomyoplasty: a new therapeutic approach for regenerating the myocardium. J Cardiovasc Nurs.

[B5] Erem C, Kandemir N, Hacihasanoglu A, Ersoz HO, Ukinc K, Kocak M (2004). Radioiodine treatment of hyperthyroidism: prognostic factors affecting outcome. Endocrine.

[B6] Metso S, Jaatinen P, Huhtala H, Luukkaala T, Oksala H, Salmi J (2004). Long-term follow-up study of radioiodine treatment of hyperthyroidism. Clin Endocrinol (Oxf).

[B7] Hadj Ali I, Khiari K, Cherif L, Ben Abdallah N, Ben Maiz H, Hajri H, Ferjaoui M Treatment of Graves' disease: 300 cases. Presse Med.

[B8] Agboola-Abu CF, Kuku SF (2003). Experience in the use of radioactive iodine therapy for hyperthyroidism in Nigerian patients. A study of twenty-two patients. West Afr J Med.

[B9] Al-Kaabi JM, Hussein SS, Bukheit CS, Woodhouse NJ, Elshafie OT, Bererhi H (2002). Radioactive iodine in the treatment of Graves' disease. Saudi Med J.

[B10] Rivkees SA, Cornelius EA (2003). Influence of Iodine-131 Dose on the Outcome of Hyperthyroidism in Children. Pediatrics.

[B11] Ahmad AM, Ahmad M, Young ET (2000). Objective estimates of the probability of developing hypothyroidism following radioactive iodine treatment of thyrotoxicosis. Eur J Endocrinol.

[B12] Nebesio TD, Siddiqui AR, Pescovitz OH, Eugster EA (2002). Time course to hypothyroidism after fixed-dose radioablation therapy of Graves' disease in children. J Pediatr.

[B13] Alexander EK, Larsen PR (2002). High dose of (131)I therapy for the treatment of hyperthyroidism caused by Graves' disease. J Clin Endocrinol Metab.

[B14] Gomez N, Gomez JM, Orti A, Gavalda L, Villabona C, Leyes P, Soler J Transient hypothyroidism after iodine-131 therapy for Grave's disease. Journal of Nuclear Medicine.

[B15] Aizawa Y, Yoshida K, Kaise N, Fukazawa H, Kiso Y, Sayama N, Hori H, Abe K (1997). The development of transient hypothyroidism after iodine-131 treatment in hyperthyroid patients with Graves' disease: prevalence, mechanism and prognosis. Clin Endocrinol (Oxf).

[B16] Shimizu K, Kumita S, Kitamura Y, Nagahama M, Kitagawa W, Akasu H, Oshina T, Kumasaki T, Tanaka S (2002). Trial of autotransplantation of cryopreserved thyroid tissue for postoperative hypothyroidism in patients with Graves' disease. J Am Coll Surg.

[B17] Reilly CP, Symons RG, Wellby ML (1986). A rat model of the 131I-induced changes in thyroid function. J Endocrinol Invest.

[B18] Reis-Filho JS, Preto A, Soares P, Ricardo S, Cameselle-Teijeiro J, Sobrinho-Simoes M (2003). p63 expression in solid cell nests of the thyroid: further evidence for a stem cell origin. Mod Pathol.

[B19] Usenko V, Lepekhin E, Lyzogubov V, Kornilovska I, Ushakova G, Witt M (1999). The influence of low doses 131I-induced maternal hypothyroidism on the development of rat embryos. Exp Toxicol Pathol.

[B20] Usenko VS, Lepekhin EA, Lyzogubov VV, Kornilovska IN, Apostolov EO, Tytarenko RG, Witt M The influence of maternal hypothyroidism and radioactive iodine on rat embryonal development: thyroid C-cells. Anat Rec.

[B21] Arufe MC, Lu M, Kubo A, Keller G, Davies TF, Lin RY Directed differentiation of mouse embryonic stem cells into thyroid follicular cells. Endocrinology.

